# Long-term outcomes of cyclosporin induction and ustekinumab maintenance combination therapy in patients with steroid-refractory acute severe ulcerative colitis

**DOI:** 10.1177/17562848231218555

**Published:** 2023-12-30

**Authors:** Francesco Vitali, Timo Rath, Entcho Klenske, Anna-Lena Vögele, Ingo Ganzleben, Sebastian Zundler, Deike Strobel, Carol Geppert, Arndt Hartmann, Markus F. Neurath, Raja Atreya

**Affiliations:** First Department of Medicine, Friedrich-Alexander-University Erlangen-Nürnberg, Erlangen, Germany; Deutsches Zentrum Immuntherapie, Friedrich-Alexander-University Erlangen-Nürnberg, Erlangen, Germany; First Department of Medicine, Friedrich-Alexander-University Erlangen-Nürnberg, Erlangen, Germany; Deutsches Zentrum Immuntherapie, Friedrich-Alexander-University Erlangen-Nürnberg, Erlangen, Germany; First Department of Medicine, Friedrich-Alexander-University Erlangen-Nürnberg, Erlangen, Germany; Deutsches Zentrum Immuntherapie, Friedrich-Alexander-University Erlangen-Nürnberg, Erlangen, Germany; First Department of Medicine, Friedrich-Alexander-University Erlangen-Nürnberg, Erlangen, Germany; Deutsches Zentrum Immuntherapie, Friedrich-Alexander-University Erlangen-Nürnberg, Erlangen, Germany; First Department of Medicine, Friedrich-Alexander-University Erlangen-Nürnberg, Erlangen, Germany; Deutsches Zentrum Immuntherapie, Friedrich-Alexander-University Erlangen-Nürnberg, Erlangen, Germany; First Department of Medicine, Friedrich-Alexander-University Erlangen-Nürnberg, Erlangen, Germany; Deutsches Zentrum Immuntherapie, Friedrich-Alexander-University Erlangen-Nürnberg, Erlangen, Germany; First Department of Medicine, Friedrich-Alexander-University Erlangen-Nürnberg, Erlangen, Germany; Deutsches Zentrum Immuntherapie, Friedrich-Alexander-University Erlangen-Nürnberg, Erlangen, Germany; Institute of Pathology, University of Erlangen-Nürnberg, Erlangen, Germany; Institute of Pathology, University of Erlangen-Nürnberg, Erlangen, Germany; First Department of Medicine, Friedrich-Alexander-University Erlangen-Nürnberg, Erlangen, Germany; Deutsches Zentrum Immuntherapie, Friedrich-Alexander-University Erlangen-Nürnberg, Erlangen, Germany; First Department of Medicine, Friedrich-Alexander-University Erlangen-Nürnberg, Ulmenweg 18, Erlangen 91054, Germany; Deutsches Zentrum Immuntherapie, Friedrich-Alexander-University Erlangen-Nürnberg, Erlangen, Germany

**Keywords:** cyclosporin, proctocolectomy, severe ulcerative colitis, sonography, ustekinumab

## Abstract

**Background:**

Effective management of patients with acute severe ulcerative colitis (ASUC) is a major challenge and there remains a paucity of available maintenance treatment options after efficacious cyclosporin induction therapy.

**Objectives:**

We investigated the long-term effectiveness and safety of cyclosporin and ustekinumab combination therapy in patients with steroid refractory ASUC.

**Design:**

Monocentric, prospective study.

**Methods:**

We included patients with steroid refractory ASUC with multiple failed prior advanced therapies, who were treated with cyclosporin and ustekinumab combination therapy.

**Results:**

Among the 11 included patients, 10 had prior failure to infliximab and 8 failed at least three previous biological therapies. The mean baseline Mayo and Lichtiger scores were 10.9 (9–12) and 13.3 (11–14), respectively. Ustekinumab was initiated 3.2 weeks (1–8) after initiation of cyclosporin treatment and combination therapy was continued for a mean of 11.5 (4–20) weeks. Clinical response was achieved in six patients at week 16 and clinical steroid-free clinical remission in five patients at week 48. Endoscopic remission was achieved in five patients at week 16 and together with histological remission in five patients at week 52. Intestinal ultrasound demonstrated mean bowel wall thickening in the sigmoid colon of 5.5 mm at baseline and 3.5 mm at week 52, respectively. Two patients had to undergo colectomy (mean 4.5 months, range 3–6) and three stopped ustekinumab therapy due to ineffectiveness. Overall, combination therapy was well tolerated.

**Conclusion:**

Combination of cyclosporin and ustekinumab therapy allowed nearly half of ASUC patients to reach clinical and endoscopic remission after 52 weeks, warranting further studies.

**Trial registration:**

Not applicable.

## Plain language summary

Effects of cyclosporin and ustekinumab combination therapy in acute severe ulcerative colitis

In this study, we looked at how to treat patients with a severe form of ulcerative colitis, a type of inflammatory bowel disease, when the usual treatments don’t work. We tested a combination of two drugs, cyclosporine and ustekinumab, to see if it could help these patients in the long term. We included eleven patients who had already tried many other treatments and didn’t get better. Most of them had also tried a drug called infliximab and had failed at least three other biological therapies. At the start, these patients were very sick, with high scores on disease activity measures. We gave them ustekinumab in addtion after a therapy with cyclosporin had been started before. The combination therapy continued for an average of almost 12 weeks. After 16 weeks, six patients showed improvement in their symptoms, and five were able to stop taking steroids. Five patients also had their colon lining looking healthy again when we looked inside with a scope after 16 weeks. And after 52 weeks, five patients had normal colon lining and healthy tissue under the microscope. Ultrasound showed that the thickness of their colon wall had decreased. Unfortunately, two patients had to have surgery to remove their colon, and three had to stop taking ustekinumab because it didn’t help them. Overall, the combination therapy was safe and well-tolerated. In conclusion, combining cyclosporin and ustekinumab helped about half of the patients with severe ulcerative colitis get better and have healthy colon lining after 52 weeks. This suggests that more research is needed to understand the benefits of this treatment in these patients.

## Introduction

Ulcerative colitis (UC) is one of the major entities of inflammatory bowel diseases (IBDs) and is characterized by intestinal inflammation that affects the rectum with continuous extension into the colon, causing lifelong morbidity. Optimized anti-inflammatory treatment of this progressive disease is essential in the optimal management of UC patients.^1^ In this context, acute severe UC (ASUC) remains a formidable medical emergency, which approximately 25% of patients will develop in their disease course, often as the index presentation. ASUC is defined as frequent bloody diarrhea with evidence of significant systemic manifestation (fever, tachycardia, anemia, and elevated inflammatory biomarkers), which necessitates hospitalization for intensive medical management or surgery. ASUC is an important marker for subsequent challenging disease course and overall up to 40% of patients will have to undergo colectomy.^2^ Various guidelines recommend intravenous corticosteroid application as first-line treatment.^3–7^ For steroid-refractory ASUC cases, surgery or second-line rescue therapy with the anti-tumor necrosis factor antibody infliximab or calcineurin inhibitors (e.g. cyclosporin) are suggested.^8–10^ Case series have also indicated potential therapeutic effectiveness of standard or high-intensity therapy with the Janus kinase (JAK) inhibitor tofacitinib or salvage therapy with upadacitinib,^11^ but these results need to be verified in corresponding trials.^12^ Cyclosporin and infliximab have proven to be similarly efficacious in randomized comparative head-to-head trials.^13,14^ As for infliximab, there are considerable rates of primary non-response, especially in patients with hypoalbuminemia, which might indicate heightened clearance of the monoclonal antibody and therefore reduced exposure to the drug substance.^15^ Cyclosporin is a non-protein-based therapy, that has demonstrated rapid onset of action and effectiveness in the setting of ASUC, but its effectiveness in maintenance treatment is limited and associated with significant toxicities. Long-term follow-up studies report up to 90% relapse rates after 3 years and colectomy rates of 58% after 7 years of cyclosporin monotherapy.^16^ Large case series have reported mortality in 1–3% of patients, serious infections in 5% of patients, and there is also a heightened risk for the occurrence of nephrotoxicity, seizures, and hypertension.^17^ It is therefore mainly used as an induction therapy in ASUC and as a sequential combination treatment with a maintenance therapeutic agent, such as azathioprine and vedolizumab.^18–23^ However, patients with previous failure to thiopurines or vedolizumab are not deemed to be suitable for cyclosporin rescue therapy due to a lack of effective maintenance treatment.

Ustekinumab is a human immunoglobulin G1 monoclonal antibody that inhibits the p40 subunit of interleukin (IL)-12 and IL-23. It has been approved for treatment of moderate-to-severe UC patients and is efficacious in inducing and maintaining remission.^24^ Furthermore, several lines of data indicate a convincing safety profile of ustekinumab, qualifying it as a potential combination partner for cyclosporin therapy.^25^ We previously reported on the first combination therapy approach with cyclosporin induction treatment, overlapping with ustekinumab maintenance treatment in a patient with ASUC.^26^ A recently published retrospective study reported effectiveness of cyclosporin and ustekinumab treatment in patients with ASUC with a median follow-up time of 9 months, but there were no endoscopic data included.^27^ Here, we report efficacy and safety of induction cyclosporin and maintenance ustekinumab combination therapy in 11 patients with ASUC, who were monitored clinically and *via* biochemical markers, sonography, endoscopy as well as histology and followed-up over 1 year, respectively.

## Methods

This prospective study was conducted at the Medical Department 1 of the University Hospital Erlangen, Germany and consecutively included all steroid-refractory ASUC adult patients that received overlapping sequential therapy with cyclosporin induction and ustekinumab maintenance therapy between August 2019 and August 2021.

Patients were included in the study if they were hospitalized due to ASUC defined by the Truelove–Witts criteria^28^ and were refractory to intravenous corticosteroid therapy at the recommended dosage (100 mg intravenous hydrocortisone four times a day or intravenous 100 mg prednisolone) for a minimum of three consecutive days. Steroid refractoriness was defined according to the ECCO (European Crohn’s and Colitis Organisation) guideline.^5^ Infectious colitis was excluded in all patients by negative stool analyses. CMV (Cytomegalovirus) superinfection was excluded in all patients with both polymerase chain reaction and immunohistochemical staining from mucosal biopsies taken prior to the commencement of cyclosporine treatment.

Patients received continuous intravenous cyclosporin treatment (2 mg/kg bodyweight) after failed steroid therapy and cyclosporin treatment responders were then switched to oral formulation cyclosporin therapy after 5 days. Cyclosporin therapy was then continued for a maximum of 6 months with regular assessment of respective trough levels and dose adjustments to maintain trough levels between 250 and 400 ng/mL. Intravenous ustekinumab therapy (6 mg/kg body weight) was initiated at a mean of 3.2 (1–8) weeks after the commencement of successful intravenous cyclosporin induction therapy. Subcutaneous ustekinumab (90 mg) maintenance therapy was administered every 6–8 weeks, depending on clinical symptoms of the individual patient.

Patients were followed-up for 12 months with regular visits to the IBD Outpatient Clinic for every ustekinumab application. Clinical symptoms, current medication, and adverse events (AEs) were assessed throughout the study period. The clinical disease activity score (partial Mayo score) was assessed at every patient visit for ustekinumab administration by the treating physician as part of the standard care procedures at our institution. Laboratory assessments [e.g. hemoglobin, C-reactive protein (CRP), albumin] were similarly done at every patient visit. Endoscopy was performed in the hospitalized patients with ASUC prior to commencement of intravenous cyclosporin treatment and at week 16 and week 52, with histological assessment of disease activity (Nancy score) in mucosal biopsies taken from the respective area of heaviest inflammation. One patient did not undergo endoscopic evaluation at week 52 due to pregnancy. Intestinal sonography examination was done prior to initiation of intravenous cyclosporin therapy during hospitalization of the patients with ASUC and at weeks 8, 16, 24, and 52 in therapy responders where the maximum bowel wall thickening of the inspected sigmoid colon was recorded. Rectal bleeding score (RBS) and stool frequency score (SFS) were separately recorded. Primary endpoints were clinical response (defined as a decrease from baseline in partial Mayo score by ⩾30% and more than or equal to three points, with a decrease in rectal bleeding subscore of ⩾1 from baseline), clinical remission according to the modified Mayo (mMyao) score (Mayo subscores for rectal bleeding of 0, for stool frequency of 0 or 1 – with more than or equal to one point decrease from baseline and an endoscopic Mayo score ⩽1), as per Selecting Therapeutic Targets in Inflammatory Bowel Disease (STRIDE)-II guidelines.^29^ Secondary endpoints were endoscopic remission and histological remission. Endoscopic remission was defined as an endoscopic Mayo score ⩽1. Histological remission was defined as a Nancy index ⩽2,^30,31^ without neutrophils in the epithelium and lamina propria. Furthermore, symptomatic remission, comprising no rectal bleeding (Mayo rectal bleeding subscore of 0) and normal to near-normal SF (Mayo SF subscore of 0 or 1) and steroid-free clinical remission, defined as absence of steroid intake 2 weeks before assessment, were other secondary endpoints. Steroid tapering was done at the treating physician’s discretion. Further secondary endpoints were biochemical remission (CRP <5 mg/L), which was assessed at weeks 8, 16, and 52 and colectomy and ustekinumab discontinuation-free survival during our follow-up period of 52 weeks.

Ultrasound response, which was not a pre-defined outcome measure of our study and was thus only reported in patients who demonstrated response to therapy as an additional response parameter, was defined as a decrease of bowel wall thickness. Although the prospective validated Milan ultrasound criteria (MUC) score is a composite score of bowel wall thickness and color Doppler pattern, which correlates with colectomy risk and endoscopy,^32^ for simplicity and reasons of standardization, we only evaluated bowel wall thickness (BWT) during follow-up. Safety was evaluated throughout the study period. All occurring AEs were recorded. The reporting of this study conforms to the Strengthening the Reporting of Observational Studies in Epidemiology statement^33^ (Supplemental File 1).

### Statistical analysis

Parametric variables are reported as mean, range, and standard deviation (SD). Graphical illustration of laboratory values, clinical scores, and Kaplan–Mayer curves were drawn with GraphPad Prism 9 (GraphPad Software, Boston, MA, USA).

## Results

Eleven consecutive patients (six males, female females, mean age: 42.3 years) with steroid-refractory ASUC and initiation of cyclosporin induction and ustekinumab maintenance therapy were included. Baseline characteristics of the hospitalized, steroid-refractory patients with ASUC prior to initiation of intravenous cyclosporin therapy are displayed in Table 1 and Supplemental Table 1. Mean disease duration was 9.3 years (range 0.5–32). The mean baseline Mayo score was 10.9 (9–12) with a mean Mayo endoscopic score of 2.7 (2–3). The mean Lichtiger score was 13.3 (11–14) and mean CRP levels were 35.7 mg/L (1.6–78.4). Altogether, 10 of the included patients had not responded to previous biological therapies, which included infliximab in all cases. One patient with low albumin levels refused offered infliximab treatment due to individual perception of potential side effects. Eight of the included patients did previously not sufficiently respond to three or more biological therapies, with one of these patients not responding to five prior biological therapies (Table 1). Altogether, seven patients failed prior vedolizumab and five patients prior tofacitinib treatment.

**Table 1.** Baseline characteristics of included patients with steroid-refractory acute severe ulcerative colitis.

Disease characteristics at baseline	
Age	42.3 years (37–66)
Sex	5 male, 6 female
Disease duration (months)	112.5 (6–384)
Current smoker	None
Montreal classification	
E1	2 (18.2%)
E2	7 (63.6%)
E3	2 (18.2%)
Mean C-reactive protein level (mg/L)	35.7 (1.6–78.4)
Mean hemoglobin level (g/dL)	12.7 (9.8–13.8)
Mean albumin level (g/dL)	34.3 (29–49.3)
Lichtiger score	13.3 (11–14)
Total Mayo score	10.9 (9–12)
Stool frequency + rectal bleeding scores	5.3 (5–6)
Mayo endoscopic subscore	2.7 (2.3)
Nancy Histological Index score	4.4 (2–5)
Previous biological therapies (*n*)	10
Five previous biological agents (*n*)	1
Four previous biological agents (*n*)	3
Three previous biological agents (*n*)	4
Previous infliximab therapy (*n*)	10
Previous vedolizumab therapy (*n*)	7
Previous tofacitinib therapy (*n*)	5

The mean time between initiation of cyclosporin and commencement of ustekinumab treatment was 3.2 weeks (1–8). Therapy with both cyclosporin and ustekinumab in combination was administered for 11.5 weeks (4–20). Oral cyclosporin therapy was continued for a mean of 2.4 months (1–5). Three patients required optimization from every 8 to every 6 weeks of subcutaneous ustekinumab (90 mg) application due to clinical signs of secondary loss of response. All patients were followed-up for 1 year after initiation of cyclosporin therapy with clinical visits at each ustekinumab administration, respectively.

**Figure 1.**
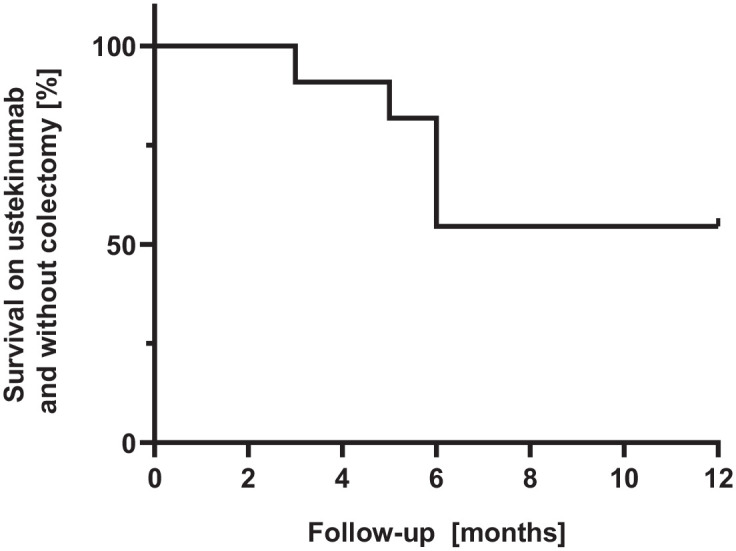
Kaplan–Meier curve of colectomy-free and ustekinumab discontinuation-free survival for all steroid-refractory ASUC patients under combination therapy with cyclosporin and ustekinumab during the study period of 52 weeks. ASUC, acute severe ulcerative colitis.

**Figure 2.**
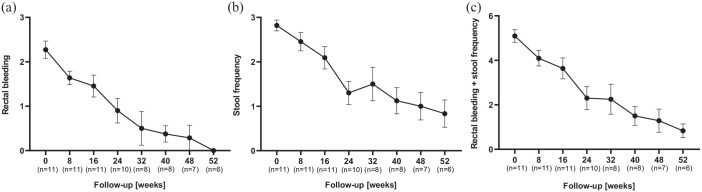
Rectal bleeding (a), stool frequency (b), and combination of rectal bleeding and stool frequency score (c) of steroid-refractory ASUC patients under combination therapy with cyclosporin and ustekinumab during the study time of 52 weeks, respectively. ASUC, acute severe ulcerative colitis.

Two patients underwent colectomy after 3 and 6 months (mean 4.5; 3–6), respectively, due to therapy-refractory disease. Ustekinumab therapy was stopped in three patients (after 2 months in one patient and after 3 months in two patients after ustekinumab induction therapy) due to ineffectiveness and need to change ongoing therapy (Figure 1). The mean RBS was 2.3 (± standard error of the mean (SEM) 0.6) at baseline, which declined to 1.6 (±0.5) at week 8, 1.5 (±0.8) at week 16, 0.9 (±0.8) at week 24, 0.5 (±1.0) at week 32, 0.4 (±0.5) at week 40, 0.3 (±0.7) at week 48, and 0.0 (±0.0) at week 52 [Figure 2(a)]. The mean SFS was 2.8 (±0.4) at baseline, which declined to 2.5 (±0.7) at week 8, 2.1 (±0.8) at week 16, 1.3 (±0.8) at week 24, 1.5 (±1.0) at week 32, 1.1 (±0.8) at week 40, 1.0 (±0.8) at week 48, and 0.8 (±0.7) at week 52 [Figure 2(b)]. The mean RBS in combination with the SFS was 5.1 (±0.9) at baseline, which declined to 4.1 (±1.1) at week 8, 3.6 (±1.5) at week 16, 2.3 (±1.6) at week 24, 2.3 (±1.8) at week 32, 1.5 (±1.1) at week 40, 1.3 (±1.3) at week 48, and 0.8 (±0.7) at week 52 [Figure 2(c)]. Symptomatic remission was therefore achieved in one patient at week 16 and five patients at week 48.

**Figure 3.**
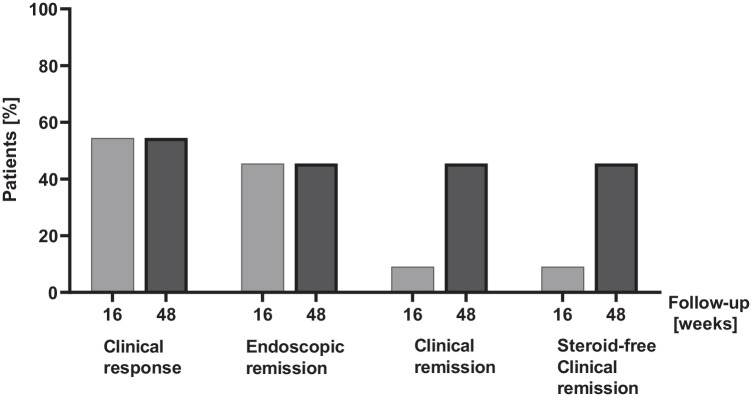
Steroid-refractory ASUC patients under combination therapy with cyclosporin and ustekinumab in clinical response, endoscopic remission, clinical remission, and steroid-free clinical remission at week 16 and week 48, respectively. ASUC, acute severe ulcerative colitis.

Clinical response was achieved in six patients at week 16 and week 48. The mean endoscopic Mayo score was 2.7 at baseline, 1.8 at week 16, and 1.2 at week 52. Endoscopic remission was achieved in five patients at week 16 and in five patients at week 52. One patient did not undergo endoscopic evaluation at week 52 due to pregnancy, but continued ustekinumab therapy due to symptomatic remission. The mean histological Nancy score was 4.4 at baseline, 2.1 at week 16, and 0.7 at week 52. Histological remission was achieved in four patients at week 16 (Nancy score 2.1, SD 1.4) and in five patients at week 52 (Nancy score 0.67, SD 0.5). Clinical remission according to the mMayo score was achieved in one patient at week 16 and five patients at week 52. All patients who achieved clinical remission at week 52 were steroid-free. One patient did not undergo endoscopic evaluation at week 52 due to pregnancy and could therefore not fulfill the criteria for clinical remission although she was in symptomatic remission (Figure 3).

The mean CRP level was 35.7 mg/L at baseline and decreased to 27.6 at week 16 and 6.9 at week 52. Biochemical remission (CRP levels <5 mg/L) was achieved in five patients at week 16 and five patients at week 52. The mean albumin level at baseline was 34.5 g/L and improved to 39.9 g/L at week 24 and 40.6 g/L at week 52. Intestinal ultrasound was performed in all patients at baseline prior to initiation of cyclosporin therapy, except two patients with isolated proctitis. Among all evaluated patients that showed response to initiated therapy, maximum mean bowel wall thickening was 5.5 mm at baseline, which decreased to 4.5 mm at week 8, 4 mm at week 16, 3.3 mm at week 24, and 3.6 mm at week 52, as further signs of therapeutic effectiveness of our initiated therapy.

AEs were reported in 5 out of 11 (45%) patients (paresthesia, myalgia, headache, nausea, hair loss) (Table 2). All observed side effects were attributed to cyclosporin treatment and cyclosporin dose reduction was necessary in all five patients. While the occurrence of AEs resolved in three patients after cyclosporin dose reduction, two patients had to discontinue cyclosporin therapy due to persistent side effects (paresthesia and hair loss). Both patients were in clinical remission at that time and continued already commenced ustekinumab therapy. No infectious AEs occurred during the time of our study.

**Table 2.** Occurrence of AEs in steroid-refractory ASUC patients treated with cyclosporin (CsA) and ustekinumab.

AE	Patients (*n*)	Onset (weeks)	Outcome
Paresthesia	4	2–6	Discontinuation CsA (*n* = 1) Resolution; CsA dose reduction (*n* = 3)
Migraine	1	2	Resolution; CsA dose reduction
Myalgia	1	2	Resolution; CsA dose reduction
Nausea	1	3	Resolution; CsA dose reduction
Loss of hair	1	6	Discontinuation CsA

AE, adverse event; ASUC, acute severe ulcerative colitis; CsA: cyclosporin A.

## Discussion

In this prospectively collected case series, we evaluated the efficacy and safety of cyclosporin induction with ustekinumab maintenance combination therapy in patients with steroid-refractory ASUC. Clinical response was achieved in over half of treated patients^6^ at week 16 and sustained till the end of our follow-up period at week 52. There was a marked decline in the rectal bleeding and SFS in the majority of patients throughout the whole study period. Clinical remission according to the mMayo score was achieved in five patients at week 48 and another patient was in symptomatic remission but an additional endoscopic examination was not performed due to pregnancy. Endoscopic remission was also achieved in five patients at weeks 16 and 52, respectively. Among treatment failures, two patients had to undergo colectomy and three patients stopped ustekinumab treatment and switched to other therapies during our respective follow-up time of 1 year, respectively. Here, it has to be considered that our cohort represents a particularly refractory collective of ASUC patients, as nearly all of them had previous unsuccessful exposure to infliximab treatment (91%) and most of the patients (81%) had received more than two previous biological substance classes and prior JAK inhibitor treatment (45%) without lasting effectiveness. As previous exposure to multiple unsuccessful biological therapies predisposes patients to diminished response to the subsequent biological, this has to be taken into account when assessing the overall good efficacy of ustekinumab maintenance treatment in this hard-to-treat patient group. Furthermore, patients were characterized with more than 9 years of disease duration and high baseline disease activity with a mean Mayo score of 10.9 and endoscopic Mayo score of 2.7.

As safety is the most important concern in combination therapy approaches, the patients were closely monitored for AEs, in particular infectious complications. Overall, cyclosporin and ustekinumab combination therapy was well tolerated. Although the narrow therapeutic index of cyclosporin and its side effect profile^6^ limit its long-term applicability, we did not observe any occurrence of infectious complications during the study period. However, cyclosporin therapy had to be discontinued in two patients with clinical remission due to cyclosporin-associated side effects (paresthesia and hair loss), which resolved after discontinuation. In line with other reports, we did not even see a correlation between cyclosporin serum levels and occurrence of the reported side effects.^34^ Ustekinumab monotherapy showed no appearance of AEs, in line with available safety data of ustekinumab treatment in UC.^24^ Doses of ustekinumab sometimes needed to be administered in shortened intervals according to clinical symptoms of patients during visits, a therapeutic strategy that was also efficaciously exerted in patients with Crohn’s disease who partially responded to ustekinumab or experienced a secondary loss of response.^34,35^

Based on our results, induction therapy in steroid-refractory ASUC patients with cyclosporin and overlapping subsequent ustekinumab maintenance therapy presents itself as a therapeutic alternative in this challenging clinical setting. As steroid-refractory ASUC patients with previous failure to infliximab are becoming more prevalent, there is a need to offer effective second-line rescue therapy options with cyclosporin induction and sequential combination therapy with a maintenance therapeutic agent. Remission induction with cyclosporin has been proven to be effective and safe according to real-word data.^36^ After cyclosporin induction treatment in steroid-refractory ASUC, maintenance therapy with azathioprine/6 mercaptopurine^37^ or vedolizumab has been proven to be effective and well tolerated.^38,39^ In our study, seven patients had already been unsuccessfully treated with thiopurines or vedolizumab. Convincing long-term efficacy and safety data of ustekinumab in UC, predispose this antibody as an ideal combination partner to cyclosporine in ASUC to maintain long-term remission with a convincing safety profile.^20,40^

A limitation of this study is the number of the patients enrolled and the inclusion of mainly biological refractory patients. Due to the small size of the patients collective, we could not identify any clinical or markers for response or failure to treatment.

The strengths of this prospective study are the density of data systematically collected at every patient visit, including sonography, as well as endoscopic and histological evaluation of the follow-up time of 1 year for each patient, respectively.

## Conclusion

Altogether, our data indicate that cyclosporin induction treatment in steroid-refractory ASUC might serve as a bridge to subsequent ustekinumab maintenance therapy. On long-term follow-up over 1 year, ustekinumab monotherapy succeeded in maintaining clinical and endoscopic remission in 45% of patients without additional requirements for steroid treatment and without continuation of oral cyclosporin treatment with a convincing safety profile. Larger studies on the evaluation of such sequential therapeutic options in ASUC are warranted.

## Supplemental Material

sj-docx-1-tag-10.1177_17562848231218555 – Supplemental material for Long-term outcomes of cyclosporin induction and ustekinumab maintenance combination therapy in patients with steroid-refractory acute severe ulcerative colitis Click here for additional data file. Supplemental material, sj-docx-1-tag-10.1177_17562848231218555 for Long-term outcomes of cyclosporin induction and ustekinumab maintenance combination therapy in patients with steroid-refractory acute severe ulcerative colitis by Francesco Vitali, Timo Rath, Entcho Klenske, Anna-Lena Vögele, Ingo Ganzleben, Sebastian Zundler, Deike Strobel, Carol Geppert, Arndt Hartmann, Markus F. Neurath and Raja Atreya in Therapeutic Advances in Gastroenterology

sj-docx-2-tag-10.1177_17562848231218555 – Supplemental material for Long-term outcomes of cyclosporin induction and ustekinumab maintenance combination therapy in patients with steroid-refractory acute severe ulcerative colitis Click here for additional data file. Supplemental material, sj-docx-2-tag-10.1177_17562848231218555 for Long-term outcomes of cyclosporin induction and ustekinumab maintenance combination therapy in patients with steroid-refractory acute severe ulcerative colitis by Francesco Vitali, Timo Rath, Entcho Klenske, Anna-Lena Vögele, Ingo Ganzleben, Sebastian Zundler, Deike Strobel, Carol Geppert, Arndt Hartmann, Markus F. Neurath and Raja Atreya in Therapeutic Advances in Gastroenterology

## Declarations

**Ethics approval and consent to participate:** Ethical review and approval were not required for the study in accordance with the local legislation and institutional requirements, as our therapeutic approach reflected standard of practice in our institution with application of approved therapeutic approaches in treating patients with ulcerative colitis. This study was performed in line with the principles of the Declaration of Helsinki.

**Consent for publication:** Not applicable.

**Availability of data and materials:** The data of findings of this study are available on request from the corresponding author (FV). The data are not publicly available due to privacy restrictions.
