# Supplementary material for: Long-term outcomes of cyclosporin induction and ustekinumab maintenance combination therapy in patients with steroid-refractory acute severe ulcerative colitis
Source: Ther Adv Gastroenterol. 2023 Dec 30;17:17562848231218555. doi: 10.1177/17562848231218555 (PMC10757791; doi:10.1177/17562848231218555)
Supplement: sj-docx-1-tag-10.1177_17562848231218555 – Supplemental material for Long-term outcomes of cyclosporin induction and ustekinumab maintenance combination therapy in patients with steroid-refractory acute severe ulcerative colitis [file sj-docx-1-tag-10.1177_17562848231218555.docx]

**Supplemental Table 1**

Key characteristics and outcomes of included patients with steroid-refractory acute sever ulcerative colitis. AZA: Azathioprin, ADA: Adalimumab, GOL: Golimumab, IFX: Infliximab, 6-MP 6 Mercaptopurin, MTX: Methotrexat, VDL: Vedolizumab, UST: Ustekinumab, MCS: Mayo partial clinical score, MES: Mayo endoscopic score, NHI: nancy histology index, BWT: bowel wall thickening, CRP: C-reactive protein

| **Pts N Sex** | **Montreal Classification** | **Disease Duration (Mo)** | **Prior biological therapy** | **Colectomy** | **Therapy failure** | **Duration of ciclosporin (days)** | **Time to ustekinumab**  **commencement (weeks)** | MCS/CRP (mg/dl)/BWT (mm)/MES/NHI  Baseline | MCS/CRP  (mg/dl)/BWT (mm)/MES/NHI  Week 16 | MCS/CRP  (mg/dl)/BWT (mm)/MES/NHI  Week 52 |
| --- | --- | --- | --- | --- | --- | --- | --- | --- | --- | --- |
| 1 F | E2 | 8 | MTX, ADA, IFX, GOL, TOFA | n | n | 5 | 3 | 7 / 1,6 / 3 / 1 / 2 | 4 / 1,9 / 3 / 1 / 0 | 2 / 1,8 / 3 / 1 / 0 |
| 2 F | E3 | 204 | None | n | n | 5 | 4 | 9 / 27,3 / 6,5 / 3 / 5 | 1 / 0,8 / 4 / 0 / 1 | 1 / 0,6 / 4 / 0 / 0 |
| 3 M | E2 | 6 | MTX, 6-MP, IFX | n | y | 5 | 2 | 8 / 37 / 8 / 3 / 5 | 4 / 14 / NA / 3 / 4 | 4 / 29,3 / NA / NA / NA |
| 4 F | E3 | 132 | AZA, ADA, VDL, IFX, UST, TOFA | n | n | 5 | 5 | 9 / 12,2 / 5 / 3 / 4 | 2 / 0,5 / 4 / 2 / 1 | 1 / 3,5 / 3 / NA / NA |
| 5 M | E3 | 96 | ADA, IFX, VDL, AZA, 6 MP, TOFA | n | n | 5 | 1 | 9 / 64 / 6 / 3 / 5 | 0 / 0,7 / 6 / 1 / 4 | 0 / 0,4 / 6 / 0 / 1 |
| 6 M | E2 | 108 | IFX, AZA, VDL, TOFA | n | y | 5 | 2 | 9 / 47 / 8 / 3 / 5 | 8 / 209 / NA / 3 / 3 | 5 / 216 / 3 / NA / NA |
| 7 M | E1 | 36 | IFX | y | y | 5 | 8 | 9 / 3,5 / 3 / 2 / 5 | 7 / 4,4 / NA / 3 / NA | NA / 1,6 / NA / NA / NA |
| 8 M | E2 | 384 | MTX, AZA, 6MP, IFX, ADA, VDL, GOL, TOFA, UST | y | y | 5 | 2 | 8 / 73 / 6 / 3 / 5 | 6 / NA / NA / NA / NA | NA / 5,5 / NA / NA / NA |
| 9 F | E2 | 96 | AZA, IFX, ADA, VDL, GOL, 6MP, TOFA | n | n | 5 | 1 | 7 / 78,4 / 7 / 3 / 5 | 1 / 16,2 / NA / 1 / 1 | 0 / 11,4 / 3 / 1 / 2 |
| 10 F | E2 | 60 | IFX, VDL, GOL | y | y | 5 | 4 | 8 / 46,3 / 6 / 3 / 4 | 5 / NA / NA / 3 / NA | NA / 40,9 / NA / NA / NA |
| 11 M | E1 | 108 | IFX, ADA, GOL, AZA, VDL | n | n | 5 | 3 | 7 / 2 / 3 / 1 / 3 | 3 / 1 / 3 / 1 / 3 | 1 / 2 / 3 / 1 / 2 |
| Median | - | 96 | - | - |  | 5 | 3 | 8 / 37 / 6 / 3 / 5 | 4 / 1,9 / 3 / 1.5 / 2 | 1 / 3,5 / 3 / 1 / 2 |
